# Vancomycin-Induced Hemolytic Anemia

**DOI:** 10.7759/cureus.39191

**Published:** 2023-05-18

**Authors:** Fakeha Siddiqui, Ahmad Cheema, Amir Kamran

**Affiliations:** 1 Internal Medicine, Camden Clark Medical Center, Parkersburg, USA; 2 Hematology-Oncology, West Virginia University, Morgantown, USA; 3 Hematology-Oncology, Charleston Area Medical Center, Charleston, USA

**Keywords:** immune-mediated hemolysis, immune-hematology, drug-induced hemolysis, hemolytic anemia, vancomycin

## Abstract

Drug-induced hemolytic anemia is rare and can occur either by an immune-mediated mechanism or a non-immune-mediated mechanism. The drugs most frequently associated with immune-mediated hemolysis are penicillins and cephalosporins. It is usually difficult to distinguish drug-induced hemolysis from other more common causes of hemolysis; therefore, a high index of clinical suspicion is required to make the diagnosis. In this case report, we present a case of vancomycin-induced immune hemolytic anemia in a 75-year-old patient who developed hemolytic anemia after starting vancomycin for joint infection. Hematological parameters improved after the discontinuation of vancomycin. Mechanism and management of drug-induced immune hemolytic anemia are also reviewed in this report.

## Introduction

Drug-induced immune hemolytic anemia (DIIHA) is a rare but well-known cause of hemolytic anemia [1]. The number of medications known to cause DIIHA has expanded over the last few years; therefore, a high index of clinical suspicions is required to make the diagnosis. The major mechanisms of DIIHA include the development of drug-dependent antibodies against the red blood cells (RBCs), alteration of RBC membrane leading to non-immunologic adsorption of proteins (e.g., IgG) onto the RBC membrane, or development of drug-independent antibodies, all leading to decreased RBC survival [2,3]. Drugs can also form immune complexes and cause hemolysis [4]. Vancomycin is a known though rare drug to induce hemolytic anemia [2,5].

Here, we present a case of vancomycin-induced immune hemolytic anemia in a 75-year-old patient.

## Case presentation

A 75-year-old male presented with progressive right knee pain associated with numbness, tingling, and weakness for five days. The patient had previously undergone hardware explantation and insertion of articulating antibiotic spacer as management for a right prosthetic joint infection with *Streptococcus dysgalactiae*. He subsequently completed a six-week treatment course of antimicrobial therapy with ceftriaxone and was subsequently placed on chronic suppressive therapy with doxycycline. He had a past medical history significant for multiple right knee total arthroplasties with spacer revisions, with two-incision and drainage procedures of posterior calf hematoma within the last three months, and vacuum-assisted closure of posterior calf wound the morning of presentation to the emergency department. The patient had grown multiple different organisms from right knee cultures in the past, including *Morganella morganii*, *Pseudomonas aeruginosa*, *Proteus*, *Enterococcus faecalis*/*avium*, anaerobes, and *Candida parapsilosis,* and recently completed IV antibiotics of piperacillin and tazobactam for 10 days.

Initial workup was remarkable for normocytic normochromic anemia, elevated sedimentation rate, and C-reactive protein (Table 1). On physical examination, the right knee was red, swollen, and tender. Due to concern of septic arthritis suspected secondary to an infected knee articulating spacer, right knee arthrocentesis was performed. Leukocyte esterase were positive, with a nucleated cell count of >35000 on synovial fluid analysis, with no crystals noted. Doxycycline was held, and the patient was started on piperacillin-tazobactam. Piperacillin-tazobactam was changed to tigecycline on day three due to leukopenia as the white cell count dropped to 2.8 x 103/uL, though hemoglobin remained stable. The patient underwent right knee explantation and revision of total knee arthroplasty articulatory spacer in the meanwhile. Blood cultures remained negative while synovial aspirate grew *Corynebacterium, *which was sensitive to vancomycin, daptomycin, and linezolid. The patient had developed eosinophilic pneumonitis on daptomycin previously, and linezolid would not have been a good choice for this patient being on multiple selective serotonin reuptake inhibitors (SSRIs) and anemia; therefore, the patient was started on vancomycin.

**Table 1:** Patient's initial investigations

Lab	Value	Reference range
White cell count	4.2 x 10^3^/uL	3.7-11 x 10^3^/uL
Hemoglobin	8.6 g/dL	13.4-17.5 g/dL
Erythrocyte sedimentation rate	97 mm/hr	0-22 mm/hr
C-reactive protein	259 mg/L	<10 mg/L
Alanine transaminase	15 U/L	8-22 U/L
Aspartate transaminase	33 U/L	8-45 U/L
Alkaline phosphatase	60 U/L	50-130 U/L
Total bilirubin	0.8 mg/dL	0.3-1.3 mg/dL
Indirect bilirubin	0.2 mg/dL	0.2-0.8 mg/dL

On the third day after switching to vancomycin, the patient's hemoglobin started to downtrend requiring multiple transfusions. Hemoglobin did not trend up despite being transfused multiple times. The patient's workup for hemolytic anemia showed elevated serum lactate dehydrogenase, undetectable haptoglobin, and elevated indirect bilirubin levels (Table 2). On direct Coombs testing, IgG antibodies were positive. Peripheral smear was negative for schistocytes, ruling out microangiopathic hemolysis. Given high suspicion for immune-mediated hemolytic anemia, the patient was started on methylprednisolone 1 mg/kg IV daily. No evidence of active bleeding was noted. CT brain was negative for intracranial bleeding. CT of the chest, abdomen, and pelvis showed no acute source of bleeding, with spleen size at the upper limits of normal. CT angiogram of the lower extremities also did not show any evidence of active bleeding. The patient was started on least incompatible blood transfusion only when required critically to avoid the development of alloantibodies.

**Table 2:** Patient's investigations at the suspicion of hemolysis

Lab	Value	Reference range
White cell count	3.3 x 10^3^/uL	3.7-11 x 10^3^/uL
Hemoglobin	5.4 g/dL	13.4-17.5 g/dL
Alanine transaminase	46 U/L	8-22 U/L
Aspartate transaminase	50 U/L	8-45 U/L
Alkaline phosphatase	172 U/L	50-130 U/L
Total bilirubin	3.9 mg/dL	0.3-1.3 mg/dL
Indirect bilirubin	3.2 mg/dL	0.2-0.8 mg/dL
Serum lactate dehydrogenase	845 U/L	125-220 U/L
Haptoglobin	undetectable	40-160 mg/dL

Given the timing of worsening anemia, no other identifiable etiology of hemolysis, and lack of response to steroids in three days, vancomycin-induced immune hemolytic anemia was the presumptive diagnosis. Vancomycin was switched to tigecycline. After discontinuing vancomycin, the hemoglobin level gradually started to trend up after three days and remained stable thereafter. Given myelosuppression with multiple drugs, and acute illness, the patient did not reach the target hemoglobin goal, but hemoglobin remained stable, and he did not require any further transfusions.

## Discussion

Drug-induced immune hemolytic anemia (DIIHA) is a well-known though rare cause of hemolytic anemia, which can result from a number of immune-mediated processes. Research shows that DIIHA occurs mainly due to the development of antibodies that are either drug-dependent or drug-independent antibodies [3,6,7].

In drug-dependent DIIHA, the drug covalently binds to proteins on the RBC membrane. If the patient develops an IgG antibody to the drug, the antibody will bind to the drug on the RBC membrane leading to phagocytosis of these drug-antibody-coated RBCs by the reticuloendothelial system resulting in extravascular hemolysis. These drug-dependent antibodies can only be detected in the presence of the inciting drug (either bound to RBCs or added to the patient's serum for testing) [3]. On the other hand, drug-independent autoantibodies do not need the presence of the drug to obtain in vitro reactions [3,8]. These autoantibodies are serologically similar to autoantibodies seen in warm autoimmune hemolytic anemia (WAIHA); therefore, the only way to diagnose DIIHA over WAIHA would be an improvement in anemia with cessation of the offending drug [7,8]

Another important mechanism is the alteration of the RBC membrane by certain drugs, which leads to the non-immunologic adsorption of proteins like IgG onto the RBC membrane. The macrophages then interact with these IgG-coated RBCs and clear them from circulation [9-11]. Some drugs can form immune complexes leading to hemolysis [1,2,3].

Vancomycin is known for causing immunologically mediated adverse reactions such as interstitial nephritis, toxic epidermal necrolysis, necrotizing cutaneous vasculitis, and red man syndrome. Isolated cases of vancomycin-associated neutropenia and thrombocytopenia have also been reported in medical literature [2]. DIIHA is a rare complication of vancomycin treatment. The drugs more commonly responsible for causing immune hemolytic anemia are cephalosporins, penicillins, platinum chemotherapy, and immune checkpoint inhibitors [12,13]. Not many cases of vancomycin-induced hemolytic anemia have been reported thus far, and only a few cases are available currently [2,14]. The case report we presented shows a rare case of hemolytic anemia secondary to vancomycin treatment within a week of starting treatment. Even though our patient was treated with multiple antibiotics in the past due to chronic history of repeated knee infections post-knee arthroplasty, hemoglobin trended down rapidly only after starting vancomycin. With no active bleeding, lack of response to packed red blood cells (PRBC) transfusions and steroids, labs consistent with hemolysis, and improvement in hemoglobin after stopping vancomycin, we believe the likely causative agent for hemolytic anemia was vancomycin. 

DIIHA should be suspected when patients present with severe complement-mediated intravascular hemolysis within hours of starting a medication, e.g., ceftriaxone, or sub-acutely with extravascular hemolysis days to weeks after exposure to a new medication [15]. Symptoms include pallor, jaundice, fatigue, dark urine, abdominal pain, or back pain. Lab abnormalities associated with hemolysis include a drop in hemoglobin/hematocrit with one or more of the typical findings such as reticulocytosis, elevated serum lactate dehydrogenase, low haptoglobin, and indirect (unconjugated) hyperbilirubinemia. A peripheral blood smear may show spherocytes or microspherocytes in case of immune hemolysis. When hemolysis is highly suspected, but the cause is unclear, a direct antiglobulin test (DAT) should be performed to determine if IgG and/or C3 are bound to the RBC membrane [15]. A positive DAT favors immune hemolysis; however, it does not differentiate between DIIHA, WAIHA, or alloimmune hemolysis from a blood transfusion. Further elution can be performed to determine if an autoantibody is present, in which case WAIHA will be favored as it is more common; however, DIIHA would not be ruled out and should be considered if there is a good temporal relationship with an offending drug [15]. 

The primary treatment of DIIHA is the discontinuation of the suspected drug. Blood counts should be monitored with expected improvement in hemoglobin within one to two weeks after stopping the drug [15]. Packed red blood cell transfusions should be provided when needed, especially in cases of severe hemolysis. The benefit of corticosteroids is unclear in this setting; however, they are usually started at the suspicion of immune-mediated hemolysis, regardless of the etiology, as noted in the Berlin case-control study of DIIHA where 85% of patients received corticosteroids [16]. Patients should be counseled on avoidance of the offending drug in the future. 

## Conclusions

DIIHA should be strongly considered when hemolytic anemia occurs after starting a new medication, especially antibiotics. Prompt diagnosis is important, and suspected drugs should be stopped as soon as possible, as delays can lead to severe complications. 
